# Digitale Gesundheitsanwendungen für depressive Störung in Deutschland: Eine narrative Übersicht über die Evidenz und Integration in die Behandlung

**DOI:** 10.1007/s00115-025-01879-7

**Published:** 2025-08-13

**Authors:** Jakob Kaminski, Felix Machleid, Caspar Wiegmann, Jan Philipp Klein, Orestis Rakitzis, Raoul Haaf, Stefanie Schreiter, Maximilian Preiß, Lukas Pezawas, Ingrid Titzler

**Affiliations:** 1 Department of Psychiatry and Neurosciences, Charité Campus Mitte, Charité – Universitätsmedizin Berlin, Corporate Member of Freie Universität Berlin, Humboldt-Universität zu Berlin, and Berlin Institute of Health, Charitéplatz 1, 10117 Berlin, Deutschland; 2 Berlin Institute of Health at Charité – Universitätsmedizin Berlin, Berlin, Deutschland; 3 Recovery Cat GmbH, Berlin, Deutschland; 4 Clinics for Psychiatry and Psychotherapy, Clinics at the Theodor-Wenzel-Werk, Berlin, Deutschland; 5 Kliniken für Psychiatrie, Psychosomatik und Psychotherapie, Universität zu Lübeck, Lübeck, Deutschland; 6 Department of Psychiatry and Psychotherapy, Medical University of Vienna, Wien, Österreich; 7 Comprehensive Center for Clinical Neurosciences and Mental Health, Medical University of Vienna, Wien, Österreich; 8 Department of Clinical Psychology and Psychotherapy, Institute of Psychology, Friedrich-Alexander-Universität Erlangen-Nürnberg, Erlangen, Deutschland; 9 PRIVATKLINIK REGENA, Bad Brückenau, Deutschland

**Keywords:** Kognitive Verhaltenstherapie, Internetbasierte Interventionen, Evidenz, Hybride Versorgung, Akzeptanz, Cognitive behavioral therapy, Internet-based interventions, Evidence, Hybrid care, Acceptance

## Abstract

**Hintergrund:**

Digitale Technologien eröffnen neue Möglichkeiten in der Behandlung psychischer Störungen. Insbesondere internet- und mobilbasierte Interventionen (IMIs) haben sich als wirksame, kosteneffiziente und zeit- und ortsunabhängige Ansätze erwiesen, die zur Verbesserung der Versorgung von Menschen mit psychischen Störungen beitragen können. In Deutschland spielen die in der Regelversorgung von allen gesetzlichen Krankenkassen erstatteten digitalen Gesundheitsanwendungen (DiGAs) eine zunehmende Rolle.

**Ziel der Arbeit:**

Ziel dieses Artikels ist es, eine narrative Übersicht zur Evidenzlage und den Herausforderungen von DiGAs für Depression im deutschen Gesundheitswesen zu bieten. Es werden die zugelassenen DiGAs vorgestellt und zentrale Studien und Entwicklungen dargestellt sowie Perspektiven für zukünftige Forschungs- und Versorgungskonzepte aufgezeigt.

**Material und Methoden:**

Die Arbeit basiert auf einer narrativen Übersicht relevanter Literatur. Ein besonderer Fokus liegt auf den durch das Bundesinstitut für Arzneimittel und Medizinprodukte (BfArM) zugelassenen DiGAs zur Behandlung von Depression und deren Einbettung in die Versorgung sowie die Akzeptanz bei Patient:innen und Behandler:innen.

**Ergebnisse:**

Insbesondere internetbasierte kognitive Verhaltenstherapie (iKVT) zeigt eine kleine bis mittlere Effektstärke bei Depressionen, die mit einer Face-to-face-Therapie vergleichbar ist (Hedge’s g = 0,43). In der Evidenz und in der Versorgung scheinen „Guided“- und „Blended-care“-Ansätze Vorteile zu bieten. Gleichzeitig ist die Nutzung von DiGAs im Vergleich zur großen Zahl der Betroffenen gering, was u. a. auf Faktoren wie fehlende Bekanntheit, komplexe Zugangswege und Verordnungsprozesse sowie Datenschutzbedenken zurückzuführen ist.

**Diskussion:**

Trotz vielversprechender Evidenz bleibt die Integration von DiGAs in bestehende Versorgungssysteme eine Herausforderung. Zukünftige Forschungsbemühungen sollten auf die Langzeitwirksamkeit, Personalisierung digitaler Lösungen und hybride Versorgungsmodelle abzielen, um Akzeptanz und Inanspruchnahme zu fördern.

**Zusatzmaterial online:**

Die Online-Version dieses Beitrags (10.1007/s00115-025-01879-7) enthält zwei Tabellen zu Charakteristika und Evidenz sowie eine Übersicht über die Begriffsdefinitionen.

## Abstract (en)

**Background:**

Digital technologies open up new opportunities in the treatment of mental health disorders. Internet-based and mobile telephone-based interventions (IMIs), in particular, have proven to be effective, cost-efficient and accessible approaches to improving the care of people with mental disorders. In Germany digital health applications (DiGAs) that are reimbursed by all public health insurances are gaining increasing importance in routine clinical practice.

**Objective:**

This article provides a narrative review of the evidence and challenges related to DiGAs for depression in the German healthcare system. The approved DiGAs are presented and key studies, developments and perspectives for future research and care concepts are discussed.

**Material and methods:**

This study is based on a narrative review of the relevant literature. The focus is on evidence-based DiGAs approved by the Federal Institute for Drugs and Medical Devices (BfArM) for the treatment of depression and their integration into routine care as well as acceptance by patients and clinicians.

**Results:**

Internet-based cognitive behavioral therapy (iCBT) in particular, demonstrate a small to medium effect strength for depression, comparable to face-to-face therapy (Hedge’s g = 0.43). Guided and blended care approaches appear to provide advantages with respect to the evidence and care; however, the utilization of DiGAs remains low, considering the large number of affected individuals, which is attributable to factors, such as limited awareness, complex access routes and prescription processes and data privacy concerns.

**Conclusion:**

Despite promising evidence, the integration of DiGAs into existing healthcare systems remains challenging. Future research efforts should focus on the long-term effectiveness, personalization of digital solutions and hybrid care models to enhance acceptance and utilization.

## Hintergrund

Internet- und mobilbasierte Interventionen (IMIs) haben sich als skalierbare (schnelle und einfache Kapazitätserhöhung), kosteneffektive [5] und zeit- und ortsunabhängige Ansätze in der Gesundheitsversorgung erwiesen. Internetbasierte kognitive Verhaltenstherapie (iKVT) hat sich in mehreren Metaanalysen als ähnlich wirksam wie konventionelle kognitive Verhaltenstherapie (KVT) gezeigt – insbesondere bei begleiteter Durchführung [1, 6, 14, 30]. Es konnte sogar eine Korrelation zwischen Dauer und dem Ansprechen auf die Behandlung festgestellt werden – ein Hinweis für eine positive Dosis-Wirkungs-Beziehung von IMIs [7]. Eine intensivere menschliche Begleitung vor und während der Intervention scheint mit einer höheren Wirksamkeit assoziiert zu sein [16]. Inzwischen zeigt eine große Metaanalyse mit über 30.000 Patient:innen eine kleine bis mittleren Effektstärke (Median g = 0,43) für iKVT [18].

Aufgrund dieser mehrfach replizierten Daten wurde in Deutschland mit dem Digitale-Versorgung-Gesetz (DVG) ein Rahmen geschaffen, um Anwendungen als digitale Therapeutika (DTx) zu verschreiben (entsprechende Regularien sind in Österreich und der Schweiz in Entwicklung). Diese digitalen Gesundheitsanwendungen (DiGAs) müssen Medizinprodukte sein und die vom Bundesinstitut für Arzneimittel und Medizinprodukte (BfArM) vorgegebenen Anforderungen an Sicherheit, Funktionstauglichkeit und Wirksamkeit erfüllen. Demgegenüber steht eine Vielzahl unzertifizierter Anwendungen. Diese fallen zwar ebenfalls unter den Begriff Mobile Health („mHealth“), erfüllen jedoch nicht die Anforderungen an Evidenz und Qualität einer DiGA. Viele dieser Anwendungen (etwa Schlaf- und Fitnesstracker oder Meditations- und Entspannungs-Apps) zielen auf die Unterstützung der psychischen Gesundheit ab, verfügen jedoch weder über eine Zulassung als Medizinprodukt noch über eine belegte Wirksamkeit.

Eine systematische Übersichtsarbeit untersuchte die Wirksamkeit von IMIs, die 2022 für Depressionen in Deutschland verfügbar waren; darunter drei DiGAs (deprexis [GAIA AG, Hamburg, Deutschland], Novego [IVP Networks GmbH, Hamburg Deuschland], Selfapy [Selfapy GmbH, Berlin, Deutschland]) und drei nicht als DiGAs gelistete IMIs (COGITO [Lara Bücker, Hamburg, Deutschland], ifightdepression [European Alliance Against Depression, Leipzig, Deutschland], moodgym [ehub Health Pty Ltd, Russell Lea NSW, Australia]; [10]). Dabei flossen 28 Studien ein, welche die IMIs im Vergleich zu aktiven (z. B. progressive Muskelrelaxation) und inaktiven (Warteliste‑)Kontrollen untersuchten. Die gepoolte Effektstärke der drei DiGAs im Vergleich zur Kontrollgruppe (KG) betrug Cohen’s d = 0,56 zum Postinterventionszeitpunkt. Es fanden sich Hinweise, dass DiGAs wirksamer sind als nichtgelistete IMIs. Die langfristigen Effekte (6–32 Wochen) fielen insgesamt schwächer aus (DiGAs: d = 0,24; nichtgelistet: d = 0,36) und zeigten keine signifikanten Unterschiede. Die Aussagekraft dieser Langzeitanalyse ist jedoch durch die Heterogenität der Nachbeobachtungszeiträume und die begrenzte Anzahl an Studien eingeschränkt, weshalb hieraus keine Handlungsempfehlung abgeleitet werden kann.

Für die Überführung von IMIs in die Regelversorgung spielt ihre Aufnahme in Leitlinien eine zentrale Rolle. Die Nationale Versorgungs-Leitlinie (NVL) Unipolare Depression empfiehlt bei einer erstmalig aufgetretenen leichten depressiven Episode den Einsatz von IMIs sogar vor einer klassischen Psychotherapie (siehe Tab. 11 NVL [2]). Darüber hinaus wird ihre Integration in Behandlungskonzepte bei klarer Indikationsstellung, therapeutischer Begleitung und Adhärenzmonitoring empfohlen. Anders als in der NVL vorgesehen, können Patient:innen in Deutschland jedoch auch ohne ärztliche Verordnung einen Zugangscode für eine DiGA direkt bei ihrer Krankenkasse anfordern – ein Paradigmenwechsel, da Kontraindikationen so lediglich auf Seiten der Krankenkassen anhand von Diagnosedaten überprüft werden können und nicht durch die eigentlichen Behandelnden. Gesetzlich sind DiGAs als eigenständige Interventionen („standalone“) konzipiert. Eine therapeutische Begleitung ist nicht verpflichtend vorgesehen – obwohl Studien und Leitlinien auf ihre Bedeutung für die Wirksamkeit hinweisen. DiGA-Anbieter können jedoch verschiedene Formen ärztlicher oder psychotherapeutischer Mitwirkung integrieren.

In diesem Beitrag sollen insbesondere die Evidenzbasis sowie die Herausforderungen bei der Implementierung von DiGAs zur Behandlung von Depressionen dargestellt werden. Ein Novum dieser Arbeit besteht darin, dass erstmals alle aktuell zugelassenen DiGAs für Depression hinsichtlich ihrer Evidenz und Integration in die Behandlung miteinander verglichen und zugleich gegenüber nicht als DiGA zertifizierten digitalen Angeboten abgegrenzt werden. Dies ermöglicht eine praxisnahe Orientierung für die klinische Anwendung und Versorgung.

## Methoden

Ziel des Artikels ist es, eine Übersicht über DIGAs zum Einsatz im Indikationsgebiet Depression zu geben. Dabei sollen insbesondere die Art der Einbindung der Behandler und die Evidenz zur Wirksamkeit betrachtet werden. Hierfür wurden systematisch alle im DiGA-Verzeichnis für die Indikationen F32.x und F33.x vorläufig und dauerhaft gelisteten DiGAs zum Zeitpunkt 14.03.2025 eingeschlossen. Abschließend wurden die im DiGA-Verzeichnis gelisteten Anwendungen in Tabellenform dargestellt und sowohl die Nachweise zur Wirksamkeit aus dem DiGA-Verzeichnis [32] als auch die zugehörigen Studien im Ergebnisteil, für jede einzelne DiGA, narrativ zusammengefasst.

## Ergebnisse

Es wurden acht DiGAs für die Indikation Depression identifiziert. HelloBetter Diabetes (GET.ON Institut für Online Gesundheitstrainings GmbH, Hamburg, Deutschland) adressiert depressive Symptome bei Menschen mit Diabetes, setzt jedoch keine formale Depressionsdiagnose voraus und wurde daher nicht in diese Analyse aufgenommen. Sechs der acht identifizierten DiGAs waren dauerhaft aufgenommen. Alle DiGAs waren sowohl für die isolierte depressive Episode als auch für die rezidivierende depressive Störung zugelassen. Überwiegend sind die DiGAs für die Anwendung bei leichter bis mittlerer Ausprägung einer depressiven Episode vorgesehen. Nur drei DiGAs wurden auch für die Anwendung bei schwergradigen depressiven Episoden ohne psychotische Symptome (International Statistical Classification of Diseases and Related Health Problems 10 [ICD-10]: F32.2, F33.2) zugelassen.

Bipolare Störungen, manische Episoden sowie das Vorliegen einer psychotischen Störung, einschließlich schizophrener Erkrankungen oder psychotischer Symptome im Rahmen affektiver Störungen, gelten als Kontraindikationen für die meisten DiGAs. Als Hauptgrund wird das potenzielle Entstehen von Wahnvorstellungen durch die verstärkte Fokussierung auf innere Prozesse angeführt. Suizidalität stellt bei allen DiGAs eine absolute Kontraindikation dar. Bis auf zwei DiGAs, die in verschiedenen Sprachen vorliegen (deprexis, und my7steps [My7steps GmbH, Wiesbaden, Deutschland]), lagen alle anderen nur in deutscher und zum Teil englischer Sprache vor. Bei einer DiGA (elona therapy Depression [Elona Health GmbH, Düsseldorf, Deutschland]) ist eine Anwendung immer zusammen mit einer vertragsärztlichen Leistung vorgesehen.

Eine tabellarische Übersicht der genannten DiGAs und zu der Anwendungsempfehlung zur Integration in die Behandlung über eine evtl. vertragsärztliche Leistung findet sich in Tabelle e1 im Onlinezusatzmaterial.

### Evidenz zur Wirksamkeit der einzelnen DiGAs

#### Deprexis

Deprexis stellt die DiGA mit der breitesten Studienlage dar und ist am längsten auf dem Markt. In einer Metaanalyse mit 12 randomisiert-kontrollierten Studien (RCTs, *N* = 2901 mit mild-moderat-schwerer depressiver Symptomatik) zeigte sich eine moderate Effektstärke in der Reduktion depressiver Symptome nach 8 bis 12 Wochen zugunsten der digitalen Intervention Deprexis (g = 0,51) zum Postinterventionszeitpunkt im Vergleich zur Kontrollbedingung (bei 9/12 Studien Warteliste mit Zugang zu „care as usual“; [27]). Die Abbruchraten in den Interventionsgruppen (IG) lagen mit einem gewichteten Durchschnitt bei 27,84 %. Vergleichbare Ergebnisse mit einem mittleren Effekt von d = 0,47 zeigte eine Metaanalyse, welche 11 Studien zu Deprexis einbezog und dabei solche mit aktiven KGs ausschloss (z. B. psychoedukative Onlineprogramme; [10]).

#### edupression.com®

In der eFICASY-Studie [20] wurden 250 Patient:innen mit leichter bis mittelgradiger Depression randomisiert entweder der IG mit Zugang zur DiGA edupression.com® (SOFY GmbH, Klosterneuburg, Österreich) oder der KG mit einer aktiven Scheinintervention zugeteilt. Hierbei handelt es sich um die einzige Studie, in der die KG eine App mit identischer grafischer Oberfläche erhielt, deren Inhalte gezielt so gestaltet waren, dass sie keine antidepressive Wirkung entfalten (z. B. Selbstaffirmationen wie Kalendersprüche, allgemeine Gesundheitstipps und Entspannungsvideos).

Die IG zeigte nach 12 Wochen eine signifikante Reduktion der Symptome (Patient Health Questionnaire 9 [PHQ-9]; d = 0,5; [20]). Zusätzlich verbesserte sich die Depressionskompetenz (D-Lit), was als Versorgungseffekt anerkannt wurde. Dies ist von Bedeutung, da edupression.com® die einzige Depressions-DiGA ist, bei der dieser Effekt offiziell anerkannt wurde. Zudem zeichnet sich edupression.com® durch die enge Einbindung der Behandler:innen aus, die Zugriff auf patientenspezifische Verlaufsdaten erhalten können. Die Drop-out-Rate lag bei 41,6 %, wobei die relativ hohe Rate mit der Drop-out-Definition zu tun hat. Patienten wurden als Drop-out klassifiziert, die eine durchschnittliche Nutzung < 2 Stimmungstagebucheinträge pro Woche oder eine Nutzungsdauer < 6 Wochen der insgesamt 12 Wochen dauernden Studie hatten.

#### elona therapy Depression

Die RCT zur Wirksamkeit von elona therapy (*N* = 283) zeigte nach 12 Wochen eine signifikante Symptomverbesserung (PHQ‑9; d = 0,62) im Vergleich zu „treatment as usual“ (TAU). Die Studie wurde präregistriert (ISRCTN11129335) und als Preprint veröffentlicht [11]. Bemerkenswert ist die enge Verzahnung mit der Behandlung durch die Mitwirkungspflicht der Behandelnden, welche ihre Patient:innen auf der Plattform anmelden und über die Freischaltung digitaler Diagnoseinstrumente und Behandlungslektionen entscheiden. Die DiGA schlägt basierend auf der Indikation ein Behandlungsprogramm vor, das von den Behandelnden an die Bedürfnisse der Patient:innen angepasst werden kann. Durch kontinuierliches Monitoring von Symptomen, Emotionen und Verhalten wird die sachgemäße Nutzung ausgewertet. Dieses enge Monitoring und die Verzahnung mit der Offline-Psychotherapie könnten mögliche Erklärungen für die geringe Drop-out-Rate (4,2 %) in der IG sein.

#### elona explore

Im Gegensatz zu elona therapy, welche nur mit Behandelnden genutzt werden kann, soll elona explore (Elona Health GmbH, Düsseldorf, Deutschland) zur Überbrückung der Wartezeit dienen. Zur vorläufigen Zulassung wurden auf der BfArM-Seite veröffentlichte Ergebnisse einer Machbarkeitsstudie herangezogen. Sie untersuchte die Wirksamkeit der digitalen Intervention in einer einarmigen, nichtrandomisierten, interventionellen, multizentrischen Studie mit 32 Teilnehmenden, von denen 31 die 10-wöchige Studie abschlossen (Drop-out-Rate 3 %). Der primäre Endpunkt war die Symptomveränderung (PHQ-9), die sich signifikant verbesserte (Mittelwert Differenz [MWD]: 8,03). Für die dauerhafte Aufnahme ist eine RCT mit 172 Teilnehmenden geplant [32].

#### MindDoc auf Rezept^1^

Für die vorläufige Aufnahme von MindDoc auf Rezept (MindDoc Health GmbH, München, Deutschland) wurde für den medizinischen Nutzennachweis eine sekundäre Datenauswertung einer RCT [12] mit verschiedenen psychischen Erkrankungen herangezogen. In die Datenauswertung flossen 262 Personen mit Depressionen (anhand von Cut-offs mit Selbstreport kategorisiert) ein. Die Intention-to-treat-Analyse (ITT) zeigte nach acht Wochen eine signifikante Reduktion der depressiven Symptomatik in der IG im Vergleich zur KG (Warteliste mit Zugang zur üblichen Behandlung, Cohens d = 0,43). Beim Erhebungszeitpunkt nach acht Wochen lagen 108 fehlende Werte (IG: 40 %; KG: 43 %) vor, nach sechs Monaten stieg der Anteil der fehlenden Werte auf 145 (IG: 62 %; KG: 48 %). Eine RCT für den Nutzennachweis und die endgültige Aufnahme ist präregistriert (DRKS00030852).

#### My7Steps-App

In der beim BfArM zum Nutzennachweis angegebenen, aber noch nicht veröffentlichten präregistrierten Studie (DRKS00025530) wurden 253 Teilnehmende eingeschlossen; acht davon (3,16 %) wurden als Drop-out klassifiziert. In der ITT-Analyse zwischen IG und KG (Warteliste) zeigte sich nach 12 Wochen (vier Wochen nach Programmende) eine statistisch signifikante Reduktion der depressiven Symptomatik, gemessen mit dem PHQ‑9 mit einem mittleren Effekt (Cohen’s d = −0,51) zugunsten der IG.

#### Novego: Depressionen bewältigen

Die im DiGA-Verzeichnis angegebene RCT hat 310 Teilnehmende mit milder bis schwerer Symptomatik untersucht und zeigt nach 12 Wochen einen signifikanten Effekt im Vergleich zur Warteliste (BDI[Beck-Depressionsinventar]-II; Hedges’ g = 0,3; DRKS00027459; [3]). Zusätzlich wird auf weitere RCTs, die in Peer-Review-Journalen veröffentlicht wurden, verwiesen. Hierbei liefert die Studie von Beiwinkel et al. [4] die überzeugendsten Ergebnisse: Von den 180 Teilnehmenden schlossen 88 die Nachuntersuchung (nach 12 Wochen) ab. Die ITT-Analyse zeigte einen signifikanten Unterschied zwischen den Gruppen hinsichtlich der Reduktion der depressiven Symptome (PHQ-9) mit einer moderaten Effektstärke (d = 0,55). Unter anderem wird auch eine Studie aufgeführt, die Depression bei Menschen mit Schizophrenie untersucht und in der auch ein positiver Effekt berichtet wird [17], zugleich wird aber Schizophrenie als Kontraindikation gelistet.

#### Selfapys-Onlinekurs bei Depression

In der im DiGA-Verzeichnis angegebenen präregistrierten (DRKS00017191) und Peer-reviewten RCT wurden 401 Teilnehmende mit depressiver Episode oder Dysthymie einer therapeutisch begleiteten (mit wöchentlichen Telefonkontakten), nichtbegleiteten oder Wartelisten-KG zugewiesen [15]. Depressionssymptome wurden u. a. mittels BDI-II zu Studienbeginn, nach sechs Wochen, nach 12 Wochen (Ende der Intervention) und nach sechs Monaten (Follow-up) erhoben. Beide IGs zeigten nach Abschluss der Intervention im Vergleich zur KG in der ITT-Analyse eine signifikante Reduktion der Symptome mit einer großen Effektstärke (begleitet: d = 1,63; nichtbegleitet d = 1,47) mit anhaltenden positiven Effekten nach sechs Monaten. In Nachuntersuchungen zeigten sich auch positive Effekte bei sekundären Endpunkten wie der psychischen und körperlichen Lebensqualität [22].

Die Belastbarkeit der Ergebnisse ist eingeschränkt, da die vergleichsweise hohe Effektstärke studienbedingt sein könnte – insbesondere durch die überraschende Symptomverschlechterung in der KG. In der Regelversorgung steht zudem eine therapeutische Begleitung durch persönliche Telefonkontakte nicht zur Verfügung. Die Abbruchrate (Drop-outs) in der IG lag bei 17,6 %.

Eine Übersicht zu der Evidenz der genannten DiGAs findet sich in Tabelle e2 im Onlinezusatzmaterial.

## Diskussion

Für fast alle im DiGA-Verzeichnis gelisteten Anwendungen liegen Wirksamkeitsnachweise aus RCTs vor. Eine Ausnahme bildet MindDoc, bei der zunächst eine Sekundäranalyse genutzt wurde und eine RCT aktuell läuft (MindDoc wurde während der Erstellung des Artikels am 25.06.2024 aus dem DiGA-Verzeichnis entfernt). Für deprexis lag bereits eine Metaanalyse vor. In der Zusammenschau zeigen sich eine erhebliche Varianz in den Effektstärken (0,3 bis 1,4) und auch große Unterschiede in der Adhärenz an die Studienprotokolle (Drop-out-Rate). Eine mögliche Erklärung können Unterschiede in den KG (Schein-App, TAU oder Warteliste) sein. Weitere mögliche Varianzquellen entstehen durch Unterschiede in der Rekrutierung (Studiensetting) sowie in der digitalen Gesundheitskompetenz der Teilnehmenden. Schließlich sind die Involvierung von Therapeuten und Behandelnden und das Vorhandensein von Mood-Monitoring weitere Einflussfaktoren auf die Adhärenz [26]. edupression.com® [20] integriert einen zusätzlichen Monitoringansatz, der eine stärkere Einbindung von Behandler:innen aus stationären und ambulanten Versorgungsstrukturen ermöglicht. Obwohl keine vertragsärztliche Leistung erforderlich ist, wird beschrieben, wie Behandler:innen auf Patientenberichte zugreifen und bei unzureichender Therapieadhärenz intervenieren können. Bei elona therapy ist eine vertragsärztliche Leistung erforderlich für die Individualisierung des Behandlungsprogramms in der App und auch für ein wiederholtes Monitoring und die Auswertung von Symptomen, Stimmung oder Verhalten. Ein systematisches Review hebt hervor, dass DiGAs klinische Wirksamkeit zeigen, jedoch in den Studien hohe Risiken für Bias bestehen aufgrund des meist herstellerseitigen Sponsorings [24]. Darüber hinaus werden als zentrale Herausforderung weitere Barrieren (wie meist fehlende Fremdsprachen, wie Farsi und Arabisch) diskutiert. Diese Lücke schließt mittlerweile die DiGA My7Steps und bietet, neben den genannten, elf Sprachen neben Deutsch an.

Wegen der vorgenannten Verzerrungen und fehlender Head-to-head-Vergleiche kann keine DiGA aufgrund ihrer Effektstärke gegenüber einer anderen bevorzugt werden. Hier sollten andere Faktoren wie die Anwendbarkeit für die Nutzer:innen, die Verfügbarkeit in unterschiedlichen Sprachen oder der Preis (Wirtschaftlichkeitsgebot) eine Rolle spielen (siehe auch Tabelle e1 im Onlinezusatzmaterial). In der narrativen Übersicht haben wir uns auf Besonderheiten der Daten fokussiert und selektiv auch Drop-out-Raten diskutiert: Starke Unterschiede in der Drop-out-Rate zwischen den DiGAs sind u. a. in unterschiedlichen Definitionen, Gesamtzahl aktiver Teilnehmender oder der Teilnahme am begleitenden Monitoring begründet. Dabei scheint der auffällig niedrige Wert (4 %) bei elona therapy möglicherweise auf die starke Involvierung der Behandler:innen, dem Rekrutierungs- und Studiensetting zurückzuführen zu sein. Evidenz aus „Real-world“-Studien liegt bisher lediglich für die DiGA deprexis vor und zeigt im Prä-post-Vergleich ohne KG hohe Effektstärken [13, 21]. Dabei gaben 20,2 % die Nutzung der DiGA frühzeitig auf. Die Drop-out-Rate der Studie ist aber nur ein Proxy für die Adhärenz an die DiGA, welche in Zukunft gesetzlich vorgegeben über die „anwendungsbegleitende Erfolgsmessung“ (AbEM) ermittelt werden muss.

Weitere Hinweise zum Nutzen bzw. zur Nutzer:innenzufriedenheit können aus App-Stores (Reviews und Ratings) abgeleitet werden [28]. DiGAs (nicht nur für Depression) zeigten im Vergleich zu nichtregulierten mHealth-Apps in Deutschland signifikant höhere App-Store-Bewertungen. Neuartige Sentimentanalysen von Nutzerbewertungen im App-Store können weitere Hinweise für die Nützlichkeit von DiGAs bieten.

Obwohl die Evidenzlage grundsätzlich positiv ist, nutzt bisher nur ein kleiner Teil der an Depressionen erkrankten Menschen DiGAs. Im Verhältnis zur sehr großen Anzahl von mehreren Millionen betroffenen Menschen in Deutschland [31] bleibt die Nutzung von DiGAs insgesamt gering: Die beiden verbreitetsten DiGAs, deprexis und Selfapy Onlinetherapie, bei Depression wurden bis September 2023 lediglich rund 40.000-mal verordnet, wenngleich die DiGAs im Bereich psychische Störungen mit 32 % den größten Anteil an eingelösten DiGAs ausmachten [9].

Vis et al. [29] identifizierten für den Einsatz von eHealth-Lösungen Barrieren wie mangelnde Schulung von Fachkräften, technische Herausforderungen und Datenschutzbedenken. Interviewte Hausärzt:innen und Therapeut:innen haben schon vor DiGA-Einführung zum Thema digitale Depressionsbehandlung gesagt, dass es u. a. eine Finanzierungslösung braucht und eine Einbettung in die bestehende Behandlung [25]. Auch nach Einführung der DiGAs zeigten sich in einer qualitativen Studie weiterhin obige Vorbehalte gegenüber DiGAs [19].

Die geringe Nutzung von DiGAs kann auf mehrere Faktoren zurückgeführt werden, darunter unzureichende Vertrautheit, mangelnde digitale Gesundheitskompetenz und komplexe Verordnungsprozesse [8]. Regulatorische Hürden, wie die „medical device regulation“ (MDR; [23]), machen es für komplexere Interventionen nicht leichter, den Weg in die Versorgung zu schaffen. Zukünftige Forschungsbemühungen sollten sich auf die Langzeitwirksamkeit, Personalisierung, Verbesserung von Adhärenz und die verbesserte Integration in die Behandlung konzentrieren.

## Fazit für die Praxis

- Internet- und mobilbasierte Interventionen (IMIs) haben das Potenzial, die Versorgung psychischer Störungen nachhaltig zu verbessern. Digitale Gesundheitsanwendungen (DiGAs) sind eine Form der Umsetzung von IMIs im deutschen Gesundheitssystem.
- Um das volle Potenzial der DiGAs auszuschöpfen, muss ihr Einsatz weiterentwickelt werden. Eine Anforderung ist die Integration der DiGAs in hybride Versorgungskonzepte, mit entsprechenden Vergütungssystemen. Dadurch könnten die aufwendige Aufklärung und Anleitung sowie die kompetente Auswertung der Nutzung kompensiert werden. Letzteres zeigt einen wichtigen Beitrag zur Wirksamkeit und Adhärenz.
- So könnte das Fach Psychiatrie und Psychotherapie es schaffen, innovative Versorgung anzubieten, ohne zu einer reinen „Gerätemedizin“ zu werden.

## Supplementary Information

Tabelle e1: Übersicht der Charakteristika der für das Indikationsgebiet Depression gelisteten Anwendungen aus dem DiGA-Verzeichnis

Tabelle e2: Übersicht der Daten zur Evidenz der für das Indikationsgebiet Depression gelisteten Anwendungen aus dem DiGA-Verzeichnis

## Footnotes

1 MindDoc wurde am 25.06.2024 aus dem DiGA-Verzeichnis entfernt.
