# Supplementary material for: Digital health applications for depressive disorders in Germany: A narrative review of the evidence and integration into treatment
Source: Nervenarzt. 2025 Aug 13;96(5):432–8. [Article in German] doi: 10.1007/s00115-025-01879-7 (PMC12411576; doi:10.1007/s00115-025-01879-7)
Supplement: Supplementary file 1 — Tabelle e1: Übersicht der Charakteristika der für das Indikationsgebiet Depression gelisteten Anwendungen aus dem DiGA-Verzeichnis [file 115_2025_1879_MOESM1_ESM.docx]

# Tabelle 1: Übersicht der Charakteristika der für das Indikationsgebiet Depression gelisteten Anwendungen aus dem DiGA-Verzeichnis (Stand 14.03.2025)

| Name | Alters gruppe | Indikation(ICD-10) | Plattform | Anwendungsempfehlung (Vertragsärztliche Mitwirkung erforderlich?) | Kosten (in Euro) | Sprachen (außer DEU) | PZN |
| --- | --- | --- | --- | --- | --- | --- | --- |
| Deprexis | >18 | F32.0, F32.1, F32.2, F33.0, F33.1, F33.2 | web | Eigenständiges Selbsthilfeprogramm in Ergänzung zu TAU (Nein) | 210 | ELL, ENG, FRA, ITA, POR, SPA, SWE, ZHO | 17265872 |
| edupression.com® | 18-65 | F32.0, F32.1, F33.0, F33.1 | web | Begleitend zu bestehender ambulanter Therapie (Nein) | 224,8 | ENG | 18458283 |
| elona explore | 18-65 | F32.0, F32.1, F32.2, F33.0, F33.1, F33.2 | mobile | Eigenständig (nein) | 535.49 | - | 19822147 |
| elona therapy Depression | 18-65 | F32.0. F32.1, F32.2, F33.0, F33.1, F33.2, F34.1 | web/mobile | Verzahnt mit Psychotherapie (ja) | 535,49 | - | 18458314 |
| MindDoc Auf Rezept [MindDoc wurde am 25.6.2024 aus dem DiGA-Verzeichnis entfernt] | >18 | F32.0, F32.1, F33.0, F33.1 | mobile | Eigenständig (nein) | 199 | ENG | 19234031 |
| My7steps App | 18-65 | F32.0, F32.1, F33.0, F33.1 | web | Eigenständig (nein) | 470,05 | ENG, ARA, FRE, ELL, ITA, FAS, POR, RUS, UKR, SPA, TUR | 18672881 |
| Novego: Depressionen bewältigen | 18-65 | F32.0. F32.1, F32.2, F33.0, F33.1, F33.2, F34.1 | web | Eigenständig (nein) | 199 | - | 17865862 |
| Selfapys Online-Kurs bei Depression | >18 | F32.0, F32.1, F33.0, F33.1 | web/mobile | Eigenständig (nein) | 217,18 | - | 16954730 |

# ARA: Arabisch, ELL: Griechisch, ENG: Englisch, FAS: Persisch, FRE: Französisch, ITA: Italienisch, POR: Portugiesisch, PZN: Pharmazentralnummer, RUS: Russisch, SPA: Spanisch, SWE: Schwedisch, TaU: Treatment as Usual (Behandlung wie Üblich), TUR: Türkisch, UKR: ukrainisch, ZHO: Chinesisch
