# Supplementary material for: Digital health applications for depressive disorders in Germany: A narrative review of the evidence and integration into treatment
Source: Nervenarzt. 2025 Aug 13;96(5):432–8. [Article in German] doi: 10.1007/s00115-025-01879-7 (PMC12411576; doi:10.1007/s00115-025-01879-7)
Supplement: Supplementary file 2 — Tabelle e2: Übersicht der Daten zur Evidenz der für das Indikationsgebiet Depression gelisteten Anwendungen aus dem DiGA-Verzeichnis [file 115_2025_1879_MOESM2_ESM.docx]

# Tabelle 2: Übersicht der Daten zur Evidenz der für das Indikationsgebiet Depression gelisteten Anwendungen aus dem DiGA-Verzeichnis (Stand 14.03.2025)

| Name | Effektstärke | Kontrollgruppe | Abbruchrate der Studienteilnahme | N (RCT) | Besonderheiten der Daten |
| --- | --- | --- | --- | --- | --- |
| Deprexis* | 0,51 (Hedges g, Divers) | Divers (vorwiegend WL) | 27,80% (real world Daten) | 2901 (12) | *Die Daten zu Deprexis stammen aus der Metaanalyse von Twomey et al, 2020^27^ |
| edupression.com® | 0,5 (Cohens d, PHQ-9, 12 Wochen, ITT) | TaU + Sham | 44% (IG), 39% (KG) | 250 (1) | Daten nicht veröffentlicht |
| elona explore | Unbekannt8.03 Punkte Reduktion, p<0,001, (Mittelwert-Differenz, PHQ-9, ITT, 10 Wochen) | TaU | 3% (einarmige Studie) | 32 (0) | Einarmig, nicht randomisiert, ANOVA bei n=32, 10 Wochen Dauer |
| elona therapy Depression | 0,62 (Cohens d, PHQ-9, 12 Wochen, ITT) | TaU | 4% (IG), 12% (KG) | 283 (1) | Daten als Preprint veröffentlicht |
| MindDoc Auf Rezept [MindDoc wurde am 25.6.2024 aus dem DiGA-Verzeichnis entfernt] | 0,43 (Cohens d, PHQ-9, ITT, 8 Wochen) | WL, TaU | 40% (IG), 43% (KG) | 262 (1) | Teilanalyse einer heterogenen Stichprobe von 1045 Patient:innen. Dropouts für Depression nicht explizit angegeben |
| My7steps App | 0,51 (Cohens d, PHQ-9, ITT) | WL, TaU | 3% (keine Differenzierung in den öffentlich zugänglichen Daten) | 253 (1) | Einarmig, literaturbasierte KG,  5 Wochen Dauer |
| Novego: Depressionen bewältigen | 0,30 (Hedges g, BDI-II, 12 Wochen, mITT) | WL | 31% (IG), 25% (KG) | 358 (1) | - |
| Selfapys Online-Kurs bei Depression | 1,41 (Cohens d, BDI-II, 12 Wochen, ITT) | WL, TaU | 23% (IG), 47% (KG) | 250 (1) | - |

# ANOVA: Analysis of Variance, BDI-II: Beck Depressions-Inventar-II, IG: Interventionsgruppe, (m)ITT: (modified) Intention-to-treat analysis, KG: Kontrollgruppe, n: Anzahl der Probanden, N: Anzahl der Studien, PHQ-9: Patient Health Questionnaire-9, RCT: Randomisierte Kontrollierte Studie, TaU: Treatment as Usual (Behandlung wie üblich), WL: Warteliste
